# 1,1′-[(1,3-Dihydroxypropane-2,2-diyl)dimethylene]dipyridinium bis­(hexa­fluoro­phosphate)

**DOI:** 10.1107/S1600536811013080

**Published:** 2011-04-13

**Authors:** Ai-lin Yuan, Chun-ling Zheng, Ling-hua Zhuang, Chang-sheng Wang, Guo-wei Wang

**Affiliations:** a Department of Light Chemical Engineering, College of Food Science and Light Industry, Nanjing University of Technology, Nanjing 210009, People’s Republic of China; b Department of Applied Chemistry, College of Science, Nanjing University of Technology, Nanjing 210009, People’s Republic of China

## Abstract

The title compound, C_15_H_20_N_2_O_2_^2+^·2PF_6_^−^, was prepared by anion exchange of two bromide ions in the ionic liquid 2,2′-bis-(pyridinium-1-ylmeth­yl)-propane-1,3-diol dibromide with potassium hexa­fluoro­phosphate. The two pyridine rings are planar (r.m.s. deviations = 0.008 and 0.00440 Å) and make a dihedral angle of 44.0 (2)°. Intermolecular O—H⋯F and C—H⋯F interactions occur. The four F atoms in each anion were refined as disordered over two sets of sites with an occupancy ration of 0.700 (19):0.300 (19).

## Related literature

For properties and applications of ionic liquids, see: Welton (1999 ▶). For dicationic ionic liquids, see: Liang *et al.* (2008 ▶); Geng *et al.* (2010 ▶); Yuan *et al.* (2010 ▶); Yang *et al.* (2010 ▶). For the synthesis of dicationic ionic liquids, see: Yuan *et al.* (2010 ▶). For bond-length data, see: Allen *et al.* (1987 ▶).
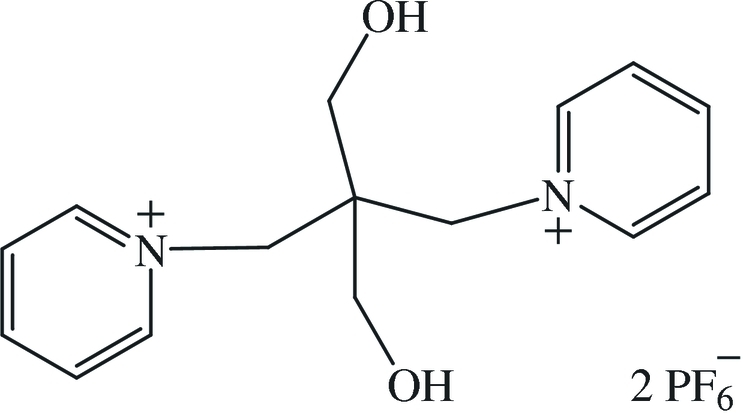


## Experimental

### 

#### Crystal data

C_15_H_20_N_2_O_2_^2+^·2PF_6_^−^
*M* *_r_* = 550.27
Monoclinic, 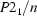

*a* = 11.955 (2) Å
*b* = 13.796 (3) Å
*c* = 12.707 (3) Å
β = 95.17 (3)°
*V* = 2087.3 (7) Å^3^
*Z* = 4
Mo *K*α radiation
μ = 0.33 mm^−1^
*T* = 293 K
0.30 × 0.20 × 0.10 mm

#### Data collection

Enraf–Nonius CAD-4 diffractometer
Absorption correction: ψ scan (North *et al.*, 1968 ▶) *T*_min_ = 0.907, *T*_max_ = 0.968
4027 measured reflections
3835 independent reflections
2564 reflections with *I* > 2σ(*I*)
*R*_int_ = 0.031
3 standard reflections every 200 reflections  intensity decay: 1%

#### Refinement

*R*[*F* ^2^ > 2σ(*F* ^2^)] = 0.053
*wR*(*F* ^2^) = 0.141
*S* = 1.04
3835 reflections
372 parameters
H-atom parameters constrained
Δρ_max_ = 0.36 e Å^−3^
Δρ_min_ = −0.26 e Å^−3^

### 

Data collection: *CAD-4 EXPRESS* (Enraf–Nonius, 1994 ▶); cell refinement: *CAD-4 EXPRESS*; data reduction: *XCAD4* (Harms & Wocadlo,1995 ▶); program(s) used to solve structure: *SHELXS97* (Sheldrick, 2008 ▶); program(s) used to refine structure: *SHELXL97* (Sheldrick, 2008 ▶); molecular graphics: *SHELXTL* (Sheldrick, 2008 ▶); software used to prepare material for publication: *SHELXTL*.

## Supplementary Material

Crystal structure: contains datablocks global, I. DOI: 10.1107/S1600536811013080/im2266sup1.cif

Structure factors: contains datablocks I. DOI: 10.1107/S1600536811013080/im2266Isup2.hkl

Additional supplementary materials:  crystallographic information; 3D view; checkCIF report

## Figures and Tables

**Table 1:** Hydrogen-bond geometry (Å, °)

*D*—H⋯*A*	*D*—H	H⋯*A*	*D*⋯*A*	*D*—H⋯*A*
C6—H6*B*⋯O1	0.97	2.47	2.831 (5)	102
C10—H10*B*⋯O2	0.97	2.44	2.796 (5)	102
O1—H1*A*⋯F8^i^	0.82	2.29	2.898 (8)	131
O2—H2*A*⋯F1	0.82	2.49	2.973 (11)	119
C1—H1*B*⋯F6	0.93	2.40	3.280 (4)	158
C11—H11*A*⋯F10^i^	0.93	2.31	3.087 (7)	141

Symmetry code: (i) 
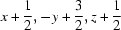
.

**Fig. 1.**
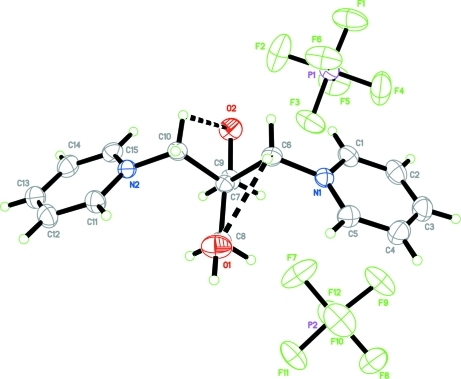
A view of the molecular structure of (I) showing the atom-numbering scheme and 30% displacement ellipsoids. Dashed lines indicte hydrogen bonds.

## supplementary crystallographic information

### Comment

Ionic liquids (ILs) have enjoyed vast research interests in recent years because of their unique physicochemical properties (Welton, 1999). Geminal dicationic ionic liquids have been shown to possess superior properties in terms of thermal stability and volatility compared to traditional ionic liquids (ILs) (Liang *et al.*, 2008). Consequently, they have been proposed as solvents in high-temperature reactions, as novel high-temperature lubricants or ultrastable separation phases (Yang *et al.*, 2010).

As part of our ongoing studies on new geminal dicationic ionic liquids (Geng *et al.*, 2010; Yuan *et al.*, 2010), we here report the crystal structure of the title compound (I).

The atom-numbering scheme of (I) is shown in Fig.1. Intramolecular C—H···O hydrogen bonds are observed between the methylene groups next to the pyridine N atoms ans the hydroxy groups. All bond lengths are within normal ranges (Allen *et al.*, 1987). The two pyridine rings are planar (r.m.s. deviations = 0.008 and 0.004 Å) and make a dihedral angle of 44.0 (2)°. (Table 1, Fig. 1).

### Experimental

A mixture of pyridine (1.98 g, 25 mmol) and 2,2-bis-(bromomethyl)-propane-1,3-diol (2.60 g, 10 mmol) was stirred vigorously at 387 K for 16 h. After cooling to room temperature, the crude product was washed with acetonitrile. The resulting solid collected by filtration was treated with water (20 ml) as well as KPF_6_ (3.68 g, 20 mmol) and the reaction mixture was stirred at room temperature for 1 h. After filtration, the white solid was washed with ethanol and dried *in vacuo* to give the title compound (I) (4.82 g, 88%). M.p. 508–510 K. Crystals of (I) suitable for X-ray diffraction were obtained by slow evaporation of a methanolic solution. ^1^H NMR (DMSO, δ, p.p.m.) 8.92 (t, 4 H), 8.68 (m, 2 H), 8.20 (m, 4 H), 5.57 (s, 2 H) 4.79 (s, 4 H), 3.16 (s, 4 H).

### Refinement

In both hexafluorophosphate groups fluorine atoms have strong oscillations, while central P atoms are fixed. Four fluorine positions in each anion have therefore been split over two positions each. All H atoms were positioned geometrically, with C—H = 0.93, 0.96 and 0.97 Å for methine, methyl, methylene H, respectively, and constrained to ride on their parent atoms, with *U*_iso_(H) = *xU*_eq_(C), where *x*= 1.5 for methyl H and *x* = 1.2 for methylene H atoms.

### Crystal data

**Table:**

C_15_H_20_N_2_O_2_^2+^·2PF_6_^−^	*F*(000) = 1112
*M**_r_* = 550.27	*D*_x_ = 1.751 Mg m^−^^3^
Monoclinic, *P*2_1_/*n*	Melting point = 508–510 K
Hall symbol: -P 2yn	Mo *K*α radiation, λ = 0.71073 Å
*a* = 11.955 (2) Å	Cell parameters from 25 reflections
*b* = 13.796 (3) Å	θ = 9–13°
*c* = 12.707 (3) Å	µ = 0.33 mm^−^^1^
β = 95.17 (3)°	*T* = 293 K
*V* = 2087.3 (7) Å^3^	Block, colorless
*Z* = 4	0.30 × 0.20 × 0.10 mm

### Data collection

**Table:**

Enraf–Nonius CAD-4 diffractometer	2564 reflections with *I* > 2σ(*I*)
Radiation source: fine-focus sealed tube	*R*_int_ = 0.031
graphite	θ_max_ = 25.4°, θ_min_ = 2.2°
ω/2θ scans	*h* = 0→14
Absorption correction: ψ scan (North *et al.*, 1968)	*k* = 0→16
*T*_min_ = 0.907, *T*_max_ = 0.968	*l* = −15→15
4027 measured reflections	3 standard reflections every 200 reflections
3835 independent reflections	intensity decay: 1%

### Refinement

**Table:**

Refinement on *F*^2^	Secondary atom site location: difference Fourier map
Least-squares matrix: full	Hydrogen site location: inferred from neighbouring sites
*R*[*F*^2^ > 2σ(*F*^2^)] = 0.053	H-atom parameters constrained
*wR*(*F*^2^) = 0.141	*w* = 1/[σ^2^(*F*_o_^2^) + (0.0676*P*)^2^ + 0.894*P*] where *P* = (*F*_o_^2^ + 2*F*_c_^2^)/3
*S* = 1.04	(Δ/σ)_max_ < 0.001
3835 reflections	Δρ_max_ = 0.36 e Å^−^^3^
372 parameters	Δρ_min_ = −0.26 e Å^−^^3^
0 restraints	Extinction correction: *SHELXL*, Fc^*^=kFc[1+0.001xFc^2^λ^3^/sin(2θ)]^-1/4^
Primary atom site location: structure-invariant direct methods	Extinction coefficient: 0.0115 (12)

### Special details

**Table:**

Geometry. All e.s.d.'s (except the e.s.d. in the dihedral angle between two l.s. planes) are estimated using the full covariance matrix. The cell e.s.d.'s are taken into account individually in the estimation of e.s.d.'s in distances, angles and torsion angles; correlations between e.s.d.'s in cell parameters are only used when they are defined by crystal symmetry. An approximate (isotropic) treatment of cell e.s.d.'s is used for estimating e.s.d.'s involving l.s. planes.
Refinement. Refinement of *F*^2^ against ALL reflections. The weighted *R*-factor *wR* and goodness of fit *S* are based on *F*^2^, conventional *R*-factors *R* are based on *F*, with *F* set to zero for negative *F*^2^. The threshold expression of *F*^2^ > σ(*F*^2^) is used only for calculating *R*-factors(gt) *etc*. and is not relevant to the choice of reflections for refinement. *R*-factors based on *F*^2^ are statistically about twice as large as those based on *F*, and *R*- factors based on ALL data will be even larger.

### Fractional atomic coordinates and isotropic or equivalent isotropic displacement parameters (Å^2^)

**Table:**

	*x*	*y*	*z*	*U*_iso_*/*U*_eq_	Occ. (<1)
C1	0.7687 (3)	0.3662 (2)	0.7039 (3)	0.0524 (8)	
H1B	0.8189	0.3301	0.6679	0.063*	
C2	0.6564 (3)	0.3615 (3)	0.6737 (3)	0.0634 (10)	
H2B	0.6302	0.3240	0.6158	0.076*	
C3	0.5825 (3)	0.4116 (3)	0.7286 (3)	0.0684 (11)	
H3A	0.5056	0.4069	0.7100	0.082*	
C4	0.6229 (3)	0.4692 (3)	0.8114 (3)	0.0659 (10)	
H4A	0.5738	0.5046	0.8492	0.079*	
C5	0.7352 (3)	0.4739 (3)	0.8378 (3)	0.0523 (8)	
H5A	0.7630	0.5135	0.8934	0.063*	
C6	0.9277 (3)	0.4206 (2)	0.8207 (2)	0.0443 (7)	
H6A	0.9569	0.3568	0.8060	0.053*	
H6B	0.9362	0.4296	0.8967	0.053*	
C7	0.9999 (2)	0.4972 (2)	0.7698 (2)	0.0366 (7)	
C8	0.9657 (3)	0.5992 (2)	0.7975 (3)	0.0491 (8)	
H8A	1.0165	0.6459	0.7703	0.059*	
H8B	0.8903	0.6127	0.7662	0.059*	
C9	0.9929 (3)	0.4856 (2)	0.6495 (2)	0.0457 (8)	
H9A	0.9157	0.4930	0.6198	0.055*	
H9B	1.0380	0.5349	0.6191	0.055*	
C10	1.1185 (2)	0.4719 (2)	0.8195 (2)	0.0416 (7)	
H10A	1.1182	0.4741	0.8958	0.050*	
H10B	1.1355	0.4058	0.8004	0.050*	
C11	1.2419 (3)	0.6103 (2)	0.8503 (3)	0.0504 (8)	
H11A	1.2049	0.6237	0.9100	0.060*	
C12	1.3296 (3)	0.6672 (3)	0.8268 (3)	0.0621 (10)	
H12A	1.3531	0.7187	0.8706	0.074*	
C13	1.3824 (3)	0.6479 (3)	0.7383 (3)	0.0649 (10)	
H13A	1.4421	0.6862	0.7213	0.078*	
C14	1.3471 (3)	0.5719 (3)	0.6748 (3)	0.0609 (10)	
H14A	1.3821	0.5587	0.6140	0.073*	
C15	1.2606 (3)	0.5161 (2)	0.7011 (3)	0.0496 (8)	
H15A	1.2369	0.4639	0.6584	0.060*	
F1	0.8561 (10)	0.3151 (7)	0.4690 (8)	0.077 (2)	0.700 (19)
F2	0.8566 (7)	0.0817 (6)	0.4416 (8)	0.095 (2)	0.700 (19)
F3	0.7337 (10)	0.1917 (9)	0.4717 (10)	0.111 (4)	0.700 (19)
F4	0.9805 (9)	0.2016 (13)	0.4446 (10)	0.156 (5)	0.700 (19)
F1'	0.886 (3)	0.3061 (18)	0.448 (2)	0.107 (10)	0.300 (19)
F2'	0.867 (3)	0.0966 (17)	0.4638 (19)	0.174 (13)	0.300 (19)
F3'	0.742 (2)	0.2191 (19)	0.455 (3)	0.111 (8)	0.300 (19)
F4'	1.0133 (16)	0.1966 (19)	0.4436 (13)	0.083 (4)	0.300 (19)
F5	0.8379 (3)	0.2040 (2)	0.33183 (18)	0.1135 (10)	
F6	0.8902 (3)	0.19322 (18)	0.57857 (18)	0.1019 (9)	
F7	0.7615 (12)	0.6122 (8)	0.5469 (9)	0.134 (4)	0.700 (19)
F8	0.5398 (6)	0.7270 (5)	0.5386 (7)	0.087 (2)	0.700 (19)
F9	0.5816 (5)	0.5722 (6)	0.5350 (8)	0.079 (2)	0.700 (19)
F10	0.7152 (4)	0.7742 (5)	0.5546 (4)	0.0600 (16)	0.700 (19)
F7'	0.783 (3)	0.620 (3)	0.5484 (16)	0.144 (13)	0.300 (19)
F8'	0.5348 (13)	0.701 (2)	0.5434 (19)	0.152 (11)	0.300 (19)
F9'	0.631 (4)	0.558 (2)	0.535 (2)	0.211 (15)	0.300 (19)
F10'	0.710 (2)	0.7468 (16)	0.5498 (16)	0.178 (15)	0.300 (19)
F11	0.6508 (2)	0.67341 (18)	0.41995 (16)	0.0884 (8)	
F12	0.6571 (2)	0.6637 (2)	0.66872 (17)	0.0994 (9)	
N1	0.8068 (2)	0.42241 (18)	0.78490 (19)	0.0401 (6)	
N2	1.2088 (2)	0.53542 (17)	0.78851 (19)	0.0391 (6)	
O1	0.9697 (2)	0.60654 (18)	0.91006 (18)	0.0683 (7)	
H1A	0.9511	0.6613	0.9264	0.102*	
O2	1.0330 (2)	0.39234 (18)	0.62617 (18)	0.0614 (7)	
H2A	1.0300	0.3852	0.5619	0.092*	
P1	0.86380 (8)	0.19842 (7)	0.45439 (7)	0.0540 (3)	
P2	0.65577 (8)	0.66706 (6)	0.54479 (6)	0.0474 (3)	

### Atomic displacement parameters (Å^2^)

**Table:**

	*U*^11^	*U*^22^	*U*^33^	*U*^12^	*U*^13^	*U*^23^
C1	0.061 (2)	0.0469 (19)	0.050 (2)	−0.0083 (17)	0.0084 (16)	−0.0058 (16)
C2	0.065 (3)	0.060 (2)	0.063 (2)	−0.017 (2)	−0.0047 (19)	−0.0065 (19)
C3	0.049 (2)	0.069 (3)	0.085 (3)	−0.007 (2)	−0.005 (2)	0.012 (2)
C4	0.055 (2)	0.071 (3)	0.073 (3)	0.006 (2)	0.0133 (19)	0.003 (2)
C5	0.056 (2)	0.055 (2)	0.0466 (19)	0.0023 (17)	0.0099 (16)	0.0003 (16)
C6	0.0475 (19)	0.0442 (18)	0.0407 (17)	0.0027 (15)	0.0015 (14)	0.0090 (14)
C7	0.0403 (17)	0.0352 (16)	0.0338 (15)	−0.0004 (13)	0.0009 (12)	0.0023 (12)
C8	0.053 (2)	0.0432 (19)	0.050 (2)	0.0045 (15)	0.0001 (15)	0.0020 (15)
C9	0.0509 (19)	0.051 (2)	0.0345 (16)	−0.0036 (15)	−0.0014 (14)	0.0063 (14)
C10	0.0473 (18)	0.0388 (17)	0.0377 (16)	−0.0003 (14)	−0.0014 (13)	0.0045 (13)
C11	0.054 (2)	0.052 (2)	0.0449 (19)	−0.0033 (16)	0.0038 (15)	−0.0099 (15)
C12	0.055 (2)	0.060 (2)	0.071 (3)	−0.0092 (18)	0.0026 (19)	−0.0154 (19)
C13	0.049 (2)	0.058 (2)	0.089 (3)	−0.0039 (18)	0.015 (2)	0.006 (2)
C14	0.051 (2)	0.071 (3)	0.063 (2)	0.0043 (19)	0.0186 (18)	−0.001 (2)
C15	0.048 (2)	0.054 (2)	0.0475 (19)	0.0074 (16)	0.0065 (15)	−0.0106 (16)
F1	0.109 (5)	0.053 (3)	0.066 (4)	0.004 (3)	−0.012 (4)	0.003 (2)
F2	0.141 (5)	0.050 (3)	0.088 (5)	0.010 (3)	−0.032 (3)	−0.037 (3)
F3	0.079 (4)	0.123 (8)	0.137 (6)	−0.047 (5)	0.039 (4)	−0.036 (6)
F4	0.027 (5)	0.255 (9)	0.188 (7)	0.012 (5)	0.012 (4)	−0.044 (6)
F1'	0.15 (2)	0.068 (11)	0.107 (14)	−0.060 (13)	0.004 (11)	−0.009 (8)
F2'	0.40 (3)	0.052 (9)	0.055 (8)	−0.032 (11)	−0.054 (11)	−0.024 (7)
F3'	0.058 (11)	0.084 (11)	0.194 (18)	0.014 (10)	0.019 (10)	0.027 (10)
F4'	0.014 (8)	0.159 (11)	0.076 (7)	0.005 (7)	0.009 (5)	−0.018 (6)
F5	0.157 (3)	0.137 (3)	0.0422 (14)	0.001 (2)	−0.0148 (15)	−0.0030 (14)
F6	0.180 (3)	0.0738 (16)	0.0473 (14)	0.0147 (17)	−0.0141 (15)	−0.0025 (11)
F7	0.088 (6)	0.116 (6)	0.196 (9)	0.047 (4)	−0.005 (5)	−0.029 (5)
F8	0.100 (6)	0.066 (3)	0.094 (4)	0.035 (3)	0.009 (3)	−0.011 (2)
F9	0.095 (4)	0.056 (4)	0.087 (4)	−0.028 (2)	0.010 (2)	−0.005 (3)
F10	0.088 (3)	0.043 (3)	0.050 (2)	−0.032 (2)	0.0108 (17)	−0.0115 (18)
F7'	0.085 (12)	0.27 (3)	0.083 (11)	0.101 (15)	0.022 (8)	0.049 (13)
F8'	0.034 (8)	0.29 (3)	0.130 (14)	−0.055 (12)	0.014 (7)	0.024 (15)
F9'	0.47 (5)	0.050 (9)	0.121 (15)	−0.05 (2)	0.08 (3)	−0.008 (8)
F10'	0.35 (4)	0.089 (12)	0.075 (8)	−0.151 (17)	0.050 (11)	−0.032 (7)
F11	0.131 (2)	0.0975 (18)	0.0378 (12)	−0.0269 (16)	0.0152 (12)	−0.0127 (11)
F12	0.151 (2)	0.109 (2)	0.0386 (13)	−0.0268 (18)	0.0083 (13)	0.0125 (12)
N1	0.0438 (15)	0.0395 (14)	0.0369 (13)	−0.0014 (12)	0.0040 (11)	0.0062 (11)
N2	0.0394 (14)	0.0395 (14)	0.0378 (14)	0.0011 (11)	−0.0003 (11)	−0.0022 (11)
O1	0.094 (2)	0.0608 (16)	0.0497 (15)	0.0114 (14)	0.0054 (13)	−0.0156 (12)
O2	0.0687 (16)	0.0663 (17)	0.0485 (14)	0.0037 (13)	0.0019 (12)	−0.0185 (12)
P1	0.0626 (7)	0.0553 (6)	0.0426 (5)	−0.0065 (5)	−0.0043 (4)	−0.0053 (4)
P2	0.0626 (6)	0.0428 (5)	0.0374 (5)	−0.0009 (4)	0.0071 (4)	−0.0009 (4)

### Geometric parameters (Å, °)

**Table:**

C1—N1	1.336 (4)	C12—C13	1.365 (5)
C1—C2	1.364 (5)	C12—H12A	0.9300
C1—H1B	0.9300	C13—C14	1.365 (5)
C2—C3	1.362 (6)	C13—H13A	0.9300
C2—H2B	0.9300	C14—C15	1.355 (5)
C3—C4	1.372 (6)	C14—H14A	0.9300
C3—H3A	0.9300	C15—N2	1.346 (4)
C4—C5	1.356 (5)	C15—H15A	0.9300
C4—H4A	0.9300	F1—P1	1.624 (10)
C5—N1	1.339 (4)	F2—P1	1.620 (9)
C5—H5A	0.9300	F3—P1	1.593 (12)
C6—N1	1.476 (4)	F4—P1	1.412 (10)
C6—C7	1.542 (4)	F1'—P1	1.51 (2)
C6—H6A	0.9700	F2'—P1	1.41 (2)
C6—H6B	0.9700	F3'—P1	1.49 (2)
C7—C8	1.516 (4)	F4'—P1	1.805 (18)
C7—C9	1.532 (4)	F5—P1	1.562 (2)
C7—C10	1.539 (4)	F6—P1	1.583 (2)
C8—O1	1.430 (4)	F7—P2	1.471 (12)
C8—H8A	0.9700	F8—P2	1.610 (6)
C8—H8B	0.9700	F9—P2	1.580 (8)
C9—O2	1.414 (4)	F10—P2	1.639 (7)
C9—H9A	0.9700	F7'—P2	1.65 (3)
C9—H9B	0.9700	F8'—P2	1.517 (19)
C10—N2	1.472 (4)	F9'—P2	1.54 (3)
C10—H10A	0.9700	F10'—P2	1.276 (19)
C10—H10B	0.9700	F11—P2	1.585 (2)
C11—N2	1.336 (4)	F12—P2	1.574 (2)
C11—C12	1.364 (5)	O1—H1A	0.8200
C11—H11A	0.9300	O2—H2A	0.8200
			
N1—C1—C2	120.2 (3)	C13—C14—H14A	120.2
N1—C1—H1B	119.9	N2—C15—C14	120.6 (3)
C2—C1—H1B	119.9	N2—C15—H15A	119.7
C3—C2—C1	119.9 (4)	C14—C15—H15A	119.7
C3—C2—H2B	120.0	C1—N1—C5	120.4 (3)
C1—C2—H2B	120.0	C1—N1—C6	118.9 (3)
C2—C3—C4	119.2 (4)	C5—N1—C6	120.5 (3)
C2—C3—H3A	120.4	C11—N2—C15	120.3 (3)
C4—C3—H3A	120.4	C11—N2—C10	119.3 (3)
C5—C4—C3	119.4 (4)	C15—N2—C10	120.4 (3)
C5—C4—H4A	120.3	C8—O1—H1A	109.5
C3—C4—H4A	120.3	C9—O2—H2A	109.5
N1—C5—C4	120.9 (3)	F2'—P1—F3'	102.4 (17)
N1—C5—H5A	119.5	F2'—P1—F1'	168.1 (19)
C4—C5—H5A	119.5	F3'—P1—F1'	89.3 (15)
N1—C6—C7	115.4 (2)	F2'—P1—F5	97.8 (10)
N1—C6—H6A	108.4	F4—P1—F5	91.1 (5)
C7—C6—H6A	108.4	F4—P1—F6	88.8 (5)
N1—C6—H6B	108.4	F5—P1—F6	179.74 (18)
C7—C6—H6B	108.4	F4—P1—F3	176.7 (8)
H6A—C6—H6B	107.5	F5—P1—F3	91.9 (5)
C8—C7—C9	109.6 (2)	F6—P1—F3	88.2 (5)
C8—C7—C10	111.9 (2)	F4—P1—F2	93.8 (8)
C9—C7—C10	110.6 (2)	F5—P1—F2	86.9 (4)
C8—C7—C6	111.5 (3)	F6—P1—F2	93.4 (4)
C9—C7—C6	111.3 (2)	F3—P1—F2	85.0 (5)
C10—C7—C6	101.7 (2)	F4—P1—F1	92.6 (8)
O1—C8—C7	108.2 (2)	F5—P1—F1	93.2 (4)
O1—C8—H8A	110.1	F6—P1—F1	86.5 (4)
C7—C8—H8A	110.1	F3—P1—F1	88.6 (6)
O1—C8—H8B	110.1	F2—P1—F1	173.6 (5)
C7—C8—H8B	110.1	F2'—P1—F4'	88.2 (16)
H8A—C8—H8B	108.4	F3'—P1—F4'	168.9 (13)
O2—C9—C7	108.4 (2)	F1'—P1—F4'	80.2 (15)
O2—C9—H9A	110.0	F10'—P2—F8'	102.6 (19)
C7—C9—H9A	110.0	F10'—P2—F9'	160 (3)
O2—C9—H9B	110.0	F8'—P2—F9'	96.8 (16)
C7—C9—H9B	110.0	F7—P2—F12	92.0 (5)
H9A—C9—H9B	108.4	F7—P2—F9	92.9 (7)
N2—C10—C7	115.2 (2)	F12—P2—F9	90.6 (4)
N2—C10—H10A	108.5	F7—P2—F11	90.1 (5)
C7—C10—H10A	108.5	F12—P2—F11	177.84 (16)
N2—C10—H10B	108.5	F9—P2—F11	89.8 (4)
C7—C10—H10B	108.5	F7—P2—F8	178.3 (6)
H10A—C10—H10B	107.5	F12—P2—F8	89.7 (3)
N2—C11—C12	120.6 (3)	F9—P2—F8	86.9 (4)
N2—C11—H11A	119.7	F11—P2—F8	88.2 (3)
C12—C11—H11A	119.7	F7—P2—F10	95.5 (6)
C11—C12—C13	119.3 (3)	F12—P2—F10	89.1 (2)
C11—C12—H12A	120.4	F9—P2—F10	171.5 (3)
C13—C12—H12A	120.4	F11—P2—F10	90.2 (2)
C14—C13—C12	119.7 (3)	F8—P2—F10	84.6 (3)
C14—C13—H13A	120.1	F10'—P2—F7'	82.6 (18)
C12—C13—H13A	120.1	F8'—P2—F7'	174.6 (16)
C15—C14—C13	119.5 (3)	F9'—P2—F7'	78 (2)
C15—C14—H14A	120.2		
			
N1—C1—C2—C3	−2.2 (6)	N2—C11—C12—C13	1.0 (5)
C1—C2—C3—C4	2.3 (6)	C11—C12—C13—C14	−0.1 (6)
C2—C3—C4—C5	−0.8 (6)	C12—C13—C14—C15	−0.6 (6)
C3—C4—C5—N1	−0.8 (6)	C13—C14—C15—N2	0.6 (5)
N1—C6—C7—C8	63.2 (3)	C2—C1—N1—C5	0.6 (5)
N1—C6—C7—C9	−59.6 (3)	C2—C1—N1—C6	175.6 (3)
N1—C6—C7—C10	−177.4 (2)	C4—C5—N1—C1	0.9 (5)
C9—C7—C8—O1	178.2 (2)	C4—C5—N1—C6	−174.0 (3)
C10—C7—C8—O1	−58.7 (3)	C7—C6—N1—C1	92.4 (3)
C6—C7—C8—O1	54.4 (3)	C7—C6—N1—C5	−92.7 (3)
C8—C7—C9—O2	175.8 (2)	C12—C11—N2—C15	−1.0 (5)
C10—C7—C9—O2	51.9 (3)	C12—C11—N2—C10	176.3 (3)
C6—C7—C9—O2	−60.4 (3)	C14—C15—N2—C11	0.2 (5)
C8—C7—C10—N2	−59.2 (3)	C14—C15—N2—C10	−177.1 (3)
C9—C7—C10—N2	63.4 (3)	C7—C10—N2—C11	95.2 (3)
C6—C7—C10—N2	−178.3 (2)	C7—C10—N2—C15	−87.5 (3)

### Hydrogen-bond geometry (Å, °)

**Table:**

*D*—H···*A*	*D*—H	H···*A*	*D*···*A*	*D*—H···*A*
C6—H6B···O1	0.97	2.47	2.831 (5)	102
C10—H10B···O2	0.97	2.44	2.796 (5)	102
O1—H1A···F8^i^	0.82	2.29	2.898 (8)	131
O2—H2A···F1	0.82	2.49	2.973 (11)	119
C1—H1B···F6	0.93	2.40	3.280 (4)	158
C11—H11A···F10^i^	0.93	2.31	3.087 (7)	141

Symmetry codes: (i) *x*+1/2, −*y*+3/2, *z*+1/2.
